# Recent advances in endometrial cancer: a review of key clinical trials from 2015 to 2019

**DOI:** 10.12688/f1000research.17408.1

**Published:** 2019-06-12

**Authors:** Lindsey M. Charo, Steven C. Plaxe

**Affiliations:** 1 Rebecca and John Moores UC San Diego Cancer Center, 3855 Health Sciences Drive #0987, La Jolla, CA, 92093-0987, USA

**Keywords:** uterine cancer, endometrial cancer

## Abstract

In the past few years, we have seen several important advances in understanding of and therapy for endometrial cancer. This review highlights key recent abstracts and publications in endometrial cancer from 2015 to 2019. We focus on clinical trials in surgical staging and the utility of sentinel lymph node mapping, adjuvant treatment for high-risk disease and HER2/neu-positive serous tumors, combination therapy for recurrent disease, molecular biology, and immunotherapy.

## Introduction

In 2019, there will be an estimated 61,880 new cases of and 12,160 deaths from endometrial cancer ^1^. It is the most common gynecologic malignancy in the US and the only gynecologic cancer with increasing incidence and mortality ^2^. Tran and Gehrig published a comprehensive F1000 Faculty Review in January 2017 ^3^. They outlined the genetic bases of endometrial cancer, novel surgical treatment, molecular targeted therapeutics, and current clinical trials. We invite you to reference their review for the groundwork of this article. Since their publication, we have seen several important advances in understanding and therapy, including surgical staging and the utility of sentinel lymph node (SLN) mapping, adjuvant treatment for high-risk disease and HER2/neu-positive serous tumors, combination therapy for recurrent disease, molecular biology, and immunotherapy. We focus on these recent advances.

## Surgical staging: sentinel lymph node evaluation

Lymph node involvement is a critical factor in determining prognosis and adjuvant therapy in endometrial cancer. As discussed in greater detail below, evidence supports a survival advantage for adjuvant chemotherapy in patients with stage III endometrial cancer; however, a similar benefit may not accrue to patients with locally advanced disease ^4– 6^. Complete staging lymphadenectomies have been associated with morbidity that impacts quality of life, including lymphedema, lymphocele formation, and neuralgia ^5, 7, 8^. Several criteria have been proposed to define patients in whom the risk is low enough to safely omit a staging lymphadenectomy. Most recently, the Korean Gynecologic Oncology Group showed that their criteria—endometrioid histology, no evidence of deep invasion or enlarged lymph nodes on magnetic resonance imaging (MRI), and pre-operative CA-125 of less than 35 units/mL—resulted in a negative predictive value of 97.1% ^9^; however, some criticize the cost and burden associated with obtaining pre-operative MRI and CA-125 ^10^. SLN biopsies offer a compromise and have been shown to reduce risks of lymphedema and lymphocele ^11^.

In 2017, the results of the Fluorescence Imaging for Robotic Endometrial Sentinel lymph node biopsy (FIRES) trial were published ^5^. This large, multicenter, prospective cohort study enrolled patients with clinical stage I endometrial cancer and determined the sensitivity and negative predictive value of SLN mapping in detecting metastatic disease compared with the gold standard complete lymphadenectomy. FIRES investigators injected indocyanine green into the cervix and performed SLN mapping followed by pelvic lymphadenectomy with or without para-aortic lymphadenectomy. Pathologists assessed SLNs for metastases by hematoxylin and eosin (H&E) staining. If the results were negative by H&E, they performed cytokeratin immunohistochemistry (IHC) ultra-staging. They found an impressive 97% sensitivity and 99.6% negative predictive value ^5^.

In the FIRES trial, approximately 14% of patients did not map and approximately 34% of patients mapped to only one side, which would necessitate approximately 48% of patients to have a lymphadenectomy due to mapping failure. Conversely, over half of patients with endometrial cancer can be spared a complete staging lymphadenectomy with SLN mapping. Some have raised concerns regarding missing isolated para-aortic metastases in SLN mapping ^12^, especially in those with the highest prevalence of para-aortic metastases (high-grade and deeply invasive tumors) ^13^. One study showed that SLN biopsies detected more metastatic nodal disease compared with selective pelvic and para-aortic lymphadenectomies ^14^. This was recently confirmed in a meta-analysis of 3,536 patients in six studies, showing higher positive pelvic nodal detection rates, no difference in para-aortic nodal detection rates, and no difference in overall recurrence or nodal recurrence rates in SLN compared with complete lymphadenectomy ^15^. Additionally, the risk of para-aortic recurrence in patients with positive pelvic nodes and unassessed para-aortic nodes is only 4% ^16^. In regard to high-risk histology, a prospective study examined women with grade 3 (endometrioid, serous, clear cell, and carcinosarcoma) endometrial cancer by SLN biopsy followed by full pelvic and para-aortic lymphadenectomy; they found 95% sensitivity and 98% negative predictive value and bilateral mapping rates of 58% and unilateral mapping rates of 40%, supporting the use of SLN in high-risk patients ^17^. Additionally, 27% of patients in the FIRES trial and 27 to 100% of patients in the six studies included in the meta-analysis had high-grade histology ^5, 15^.

In order to adequately evaluate nodal status, patients who do not map to one or both sides should have unilateral or bilateral complete lymphadenectomies, respectively. Additionally, all enlarged or suspicious lymph nodes should be removed, regardless of SLN mapping ^18^. According to the Society of Gynecologic Oncology (SGO) Clinical Practice Statement, individual surgeons should consider performing full lymphadenectomy while their personal experience is accrued (20 to 30 cases) to ensure that they, and their pathologists, demonstrate acceptable sensitivity and negative predictive value. The decreased morbidity, high sensitivity, and high negative predictive value make SLN evaluation an appealing and standard option, which is supported by both the National Comprehensive Cancer Network (NCCN) and the SGO; however, some surgeons may choose to limit its use to intermediate-risk populations until further data accrue. Additionally, data to guide adjuvant treatment (that is, adding extended-field radiation) in patients with positive pelvic lymph nodes with unknown para-aortic nodal status are needed.

## Adjuvant treatment for high-risk endometrial cancers

Most endometrial cancers will be cured with surgery alone. However, many advanced-stage and some early-stage cancers will recur. Multiple studies have characterized the risk of post-surgical recurrence and tried to identify adjunctive therapies to reduce it. High-risk factors include tumor grade, tumor size, cell type, depth of myometrial invasion, presence of lymphovascular space invasion (LVSI), and stage ^13, 19– 21^. Because of randomized controlled trials and retrospective series comparing adjuvant chemotherapy with radiation therapy, there has been optimism that chemotherapy may reduce the risk of distant recurrence and improve survival for patients with high-risk disease. However, as outlined below, chemotherapy has shown improvement in overall survival (OS) in advanced-stage disease but not in high-risk, early-stage patients. Radiation therapy is known to be quite effective in achieving local control, but improvement in OS has not been realized ^6, 19, 22, 23^. Recently, the results of three important trials that evaluated combined radiation and chemotherapy aimed at reducing rates of both local and distant recurrence were presented: the results of GOG 249 were published in the *Journal of Clinical Oncology* in April 2019 ^24^, Gynecologic Oncology Group (GOG) protocol 258 was presented in abstract form ^25^, and the results of Post-Operative Radiation Therapy in Endometrial Carcinoma 3 (PORTEC 3) were published in *Lancet Oncology* in March 2018 ^26^. GOG 249 investigated the role of adjuvant chemotherapy and vaginal brachytherapy in early-stage, high-risk patients ^24^, whereas GOG 258 ^24– 26^ and PORTEC 3 ^24– 26^ examined the role of combined chemotherapy and radiation in higher-risk cohorts. Table 1 outlines the details of these and other key trials that guide adjuvant therapy in high-intermediate and high-risk endometrial cancer.

**Table 1.** Key trials guiding adjuvant therapy in high-intermediate and high-risk endometrial cancer.

Trial Years of accrual Number of patients assessed First author Publication year	Eligibility		LN assessed	Arms	Aims	Outcomes		Conclusions
PORTEC 1990–1997 N = 714 Creutzberg *et al*. (2000) ^22^	Stage I: IB ^a^ G2–3 ^b^ IC G1–2 S/CC		No	Observation versus WPRT (46 Gy)	Primary: Locoregional recurrence and death Secondary: Treatment- related morbidity and survival after relapse	-5-year locoregional recurrence: 14% obs versus 4% WPRT ( *P* <0.001) -5-year OS: 85% obs versus 81% WPRT ( *P* = 0.31) -Cancer-related death: 6% obs versus 9% WPRT ( *P* = 0.37) -Treatment-related complications: 6% obs versus 25% WPRT ( *P* <0.0001) -2-year survival after recurrence: 79% after vaginal versus 21% after pelvic/distant recurrence -15-year follow-up: locoregional recurrence 15.5% obs versus 6% WPRT ^23^		-Adjuvant RT improves locoregional control, not OS -Limit adjuvant RT to patients age > 60 with G3 and less than half myometrial invasion or any grade with outer half myometrial invasion -Avoid adjuvant RT if age < 60 or G2 with superficial invasion (risk locoregional recurrence < 5%) -15-year follow-up study: confirms. Limit adjuvant pelvic RT to HIR cohort ^23^
GOG 99 1987–1995 N = 392 Keys *et al*. (2004) ^19^	“Intermediate risk:” IB IC II (occult) ^a^		Yes	Observation versus WPRT (50.4 Gy)	Primary: Toxicity, date and location of recurrence, OS	-Authors defined a HIR group by age and number of RFs: RF: grade 2–3, LVSI, and outer 1/3		-Adjuvant radiation decreases risk of recurrence, but not OS -Limit adjuvant pelvic radiation to patients who fit HIR group
						Age	# RF	
						<50	3	
						50–69	2	
						>70	1	
						In all patients: -2-year cumulative incidence of recurrence: 12% obs versus 3% WPRT (RH: 0.42, *P* = 0.007) -18 versus 3 vaginal recurrences -OS: 86% obs versus 92% WPRT (RH: 0.86, *P* = 0.557) In the HIR group: -2-year recurrence: 26% obs versus 6% WPRT		
PORTEC-2 2002–2006 N = 427 Nout *et al*. (2010) ^27^	Age > 60 IB G3 IC G1 or 2 ^a^ IIA (any age, exclude G3 with outer half invasion) Excluded S/CC		No	EBRT (46 Gy in 23 fx) versus VCB (21 Gy HDR in 3 fx or 30 Gy LDR)	Non-inferiority trial Primary: Vaginal recurrence	-5-year vaginal recurrence rate: 1.8% VCB versus 1.6% EBRT (HR 0.78, 95% CI 0.17–3.49, *P* = 0.74) -5-year locoregional relapse: 5.1% VCB versus 2.1% EBRT (HR 2.08, 95% CI 0.71–6.09, *P* = 0.17) -OS: 84.8% VCB versus 79.6% EBRT (HR 1.17, 95% CI 0.69–1.98; *P* = 0.67) -Rates grade 1–2 gastrointestinal toxicity: 12.6% VCB versus 53.8% WPRT		-VCB is non-inferior to WPRT in this HIR group, with fewer gastrointestinal toxic effects -Of note, LVSI not considered
GOG 249 2009–2013 N = 601 Randall *et al*. (2019) ^24^	I with HIR criteria: RF: Outer 1/2, Grade 2 or 3, LVSI		Optional: 89% assessed If not assessed, required imaging (CT or MRI) to rule out enlarged lymph nodes	Pelvic RT (4 field or IMRT, 45 to 40.5 Gy over 5–6 weeks) Additional VCB optional for S/CC or II) versus VCB (HDR 6–7 Gy at 0.5 cm depth x 3 fx, HDR 10–10.5 Gy at vaginal surface ×3 fx, or 6 Gy at vaginal surface × 5 fx or LDR 65–70 Gy at vaginal surface in 1–2 fx), followed by carboplatin AUC 6/ paclitaxel 175 mg/m ^2^ Q 21 days × 3 cycles (VCB/C)	Primary: RFS Secondary: OS, patterns of failure, toxicity	-60-month RFS: 0.76 RT (95% CI 0.70–0.81) versus 0.76 VCB/C (95% CI 0.70–0.81) -60-month OS: 0.87 RT (95% CI 0.83–0.91) versus 0.85 VCB/C (95% CI 0.81–0.90) -No difference in vaginal or distant failures -5-year pelvic and para-aortic nodal failures: 9% VCB/C versus 4% RT, HR 0.47 -5-year vaginal recurrence: 2.5% versus 2.5% -5-year distant recurrence: 18% versus 18% -Toxicity: > Grade 3 acute toxicity: 11% RT versus 64% VCB/C > Grade 3 late toxicity: 13% RT versus 12% VCB/C -At 11 weeks, VCB/C arm had 3.7 points lower (98.3% CI -5.9–1.6, *P* <0.001) on FACIT fatigue subscale score than RT arm. RT arm returned to baseline at 11 weeks and VCB/C arm returned to baseline at 8 months -VCB/C arm reported more neurotoxicity than RT arm, however returned to baseline at 14 months		-VCB/C did not improve RFS or OS compared with RT -In subgroup analysis (including serous and clear cell), VCB/C did not improve RFS or OS -Pelvic and para-aortic nodal failures more common in VCB/C -Acute mild/moderate toxicities greater in VCB/C arm, while late toxicities similar versus RT -Pelvic radiation preferred for high-risk, early-stage endometrial carcinoma
	Age	# RF						
	<50	3						
	50–69	2						
	>70	1						
	II I–II serous or clear cell with negative washings (15% serous, 5% CC)							
**Locally advanced**							
GOG 122 1992–2000 N = 396 Randall *et al*. (2006) ^6^	III/IV (post- op residual disease <2 cm)		Optional: 86% assessed	WART (30 Gy in 20 fx, with a 15-Gy boost) versus doxorubicin 60 mg/m ^2^ and cisplatin 50 mg/m ^2^ Q 3 weeks × 7 cycles, followed by 1 cycle of cisplatin (AP)	Primary: PFS Secondary: OS	Stage-adjusted progression: HR 0.71, 95% CI 0.55 to 0.91; *P* <0.01 Local recurrence: 13% WART versus 18% AP Distant recurrence: 38% WART versus 32% AP 5-year stage-adjusted disease-free survival: 50% CT versus 38% WART 5-year stage-adjusted OS: 55% AP versus 42% WART		-Chemotherapy improved PFS and OS compared with WART
PORTEC-3 2006–2013 N = 660 de Boer *et al*. (2018) ^26^	IA G3 with LVSI IB G3 II III I–III S or CC (IA S or CC required to have myometrial invasion)		Optional: 58% assessed	Pelvic RT (48.6 Gy in 1.8 Gy fx) versus combination chemotherapy and radiation (CTRT) (cisplatin 50 mg/m ^2^ weeks 1 and 4 of RT, followed by carboplatin AUC 5 and paclitaxel 175 mg/m ^2^ Q 3 weeks × 4 cycles)	Primary: OS and FFS	-5-year OS: 81.8% CTRT versus 76.7% RT (HR 0.76, 95% CI 0.54–1.06; *P* = 0.11) -5-year FFS: 75.5% CTRT versus 68.8% RT (HR 0.71, 95% CI 0.53–0.95; *P* = 0.022) > Grade 3 adverse events: 60% CTRT versus 12% RT Subgroup analysis of stage III patients: -5-year FFS 69.3% CTRT versus 58% RT (HR 0.66, 95% CI 0.45–0.97) -5-year OS 78.7% CTRT versus 69.8% RT (HR 0.71, 95% CI 0.45–1.11)		-CTRT did not improve OS, although it did increase FFS -Subgroup analysis of stage III: improved 5-year FFS (HR 0.66) and trend toward improved OS
GOG 258 2009–2014 N = 733 Matei *et al*. (2017) (abstract) ^25^	III–IVA (<2 cm residual disease) I–II S/CC with positive washings		Optional: (% N/A)	Cisplatin 50 mg/m ^2^ intravenous D1 and D29 plus volume- directed RT (45 Gy ± brachytherapy) followed by carboplatin AUC 5/ paclitaxel 175 mg/m ^2^ Q 21 days × 4 cycles with G-CSF support (CRT) versus Carboplatin AUC 6/ Paclitaxel 175 mg/m ^2^ Q 21 days × 6 cycles (CT)	Primary: RFS Secondary: survival, toxicity, quality of life	-Vaginal recurrence: 3% CRT versus 7% CT (HR = 0.36, 95% CI 0.16–0.82) -Pelvic and para-aortic node recurrence: 10% CRT versus 21% CT (HR 4.2, 95% CI 0.28–0.66) -Distant recurrence: 28% CRT versus 21% CT, HR 1.36, 95% CI 1–1.86) -6-year recurrence-free survival: 35.7% CRT versus 38.0% (HR 0.9, 95% CI 0.74–1.1) > Grade 3 toxicity: 58% CRT versus 63% CT -Survival and quality of life endpoints not yet reported		-Chemoradiation did not improve RFS compared with chemotherapy (HR = 0.9, 95% CI 0.74–1.1) -More acute toxicities in CRT versus CT -Fewer vaginal, pelvic, and para- aortic failures in CRT versus CT -Distant recurrences more common in CRT than CT

^a^1998 FIGO (International Federation of Gynaecology and Obstetrics) surgical staging: IA: tumor limited to endometrium, IB: tumor invasion less than one half of the myometrium, IC: tumor invasion more than half of the myometrium. II: cervical involvement (included endocervical glandular involvement and cervical stromal invasion). ^b^Grade 1 (G1) ≤ 5% non-squamous or non-morular solid growth pattern; grade 2 (G2): 6–50% non-squamous or non-morular solid growth pattern; and grade 3 (G3): >50% non-squamous or non-morular solid growth pattern. AP, doxorubicin/cisplatin; AUC, area under the curve; CC, clear cell; CI, confidence interval; CRT, conformal radiation therapy; CT, computed tomography; CTRT, chemotherapy and radiation therapy; EBRT, external beam radiation therapy; FACIT, Functional Assessment of Chronic Illness Therapy; FFS, failure-free survival; fx, fraction; GOG, Gynecologic Oncology Group; Gy, gray; obs, observation; HDR, high-dose radiation therapy; HIR, high-intermediate risk; HR, hazard ratio; IMRT, intensity-modulated radiation therapy; LDR, low-dose radiation therapy; LN, lymph node; LVSI, lymphovascular space invasion; MRI, magnetic resonance imaging; N/A, not available; OS, overall survival; PFS, progression-free survival; PORTEC, Post-Operative Radiation Therapy in Endometrial Carcinoma; Q, every; RF, risk factor; RFS, recurrence-free survival; RH, relative hazard; RT, radiation therapy; S, serous; VCB, vaginal cuff brachytherapy; WART, whole abdominal radiation therapy; WPRT, whole pelvic radiation therapy.

### Stage I and II endometrioid endometrial cancers

Certain subgroups of early-stage endometrial cancer have a high risk of recurrence. Based on the risk factors identified in GOG 33, a subgroup was identified in GOG 99 as “high-intermediate risk” ^13^. This cohort accounted for two thirds of recurrences and cancer-related deaths. From these studies, the GOG 249 investigators identified a population of high-risk, early-stage endometrial cancer patients who may benefit from aggressive adjuvant therapy ^24^. Investigators compared vaginal cuff brachytherapy combined with chemotherapy to pelvic radiation. This was the first GOG study to include intensity-modulated radiation therapy. After a median follow-up of 53 months, they found that chemoradiation did not improve progression-free survival (PFS) and patients receiving chemotherapy experienced more adverse events. There were no differences in vaginal or distant failure rates; however, pelvic and para-aortic nodal failures were more common among patients who received vaginal cuff brachytherapy with chemotherapy. Of note, a subset analysis of patients with serous or clear cell tumors did not find a benefit in PFS or OS with vaginal cuff brachytherapy combined with chemotherapy.

The PORTEC 3 trial, discussed in further detail below, also included high-risk, early-stage endometrioid endometrial cancers (stage I grade 3 endometrioid cancers with deep myometrial invasion or LVSI and stage II endometrioid cancers). This study compared chemoradiation to whole pelvic radiation and did not find significant improvement in failure-free survival (FFS) (hazard ratio [HR] 0.79, 95% confidence interval [CI] 0.47–1.33) or OS (HR 0.77, 95% CI 0.49–1.21) to justify the addition of chemotherapy in stage I or II endometrioid cancers ^26^.

Given increased toxicity without PFS or OS benefit, we would recommend against chemotherapy for high-risk stage I and II endometrioid endometrial cancers. Radiation should be considered for local control. Given the results of the PORTEC trials ^22, 23, 26, 27^, it would be reasonable to offer external beam radiation therapy (EBRT) for grade 3 endometrioid cancers with deep myometrial invasion or LVSI or both. Other high-intermediate risk endometrioid cancers can be treated with adjuvant vaginal cuff brachytherapy ^22, 23, 27^.

### Advanced endometrioid endometrial cancers

Defining the optimal post-operative adjuvant treatment for advanced endometrial cancer remains challenging. There is increased risk of distant failure in patients who receive radiation alone and increased pelvic failures in patients who receive chemotherapy alone. GOG 122 showed a PFS and OS benefit with chemotherapy (doxorubicin 60 mg/m ^2^ and cisplatin 50 mg/m ^2^ every three weeks for seven cycles, followed by one cycle of cisplatin) compared with whole abdomen radiation therapy in patients with stage III to IVA with less than 2 cm post-operative residual disease ^6^.

Given the concern for increased pelvic relapse with adjuvant chemotherapy alone in high-risk endometrial cancer, the phase II RTOG-9708 trial assessed the toxicity of adjuvant combined chemoradiation and chemotherapy in grade 2 or 3 endometrial adenocarcinoma with outer one half myometrial invasion, cervical stromal invasion, or pelvic-confined extrauterine disease ^28^. The trial showed promising locoregional control (4-year disease-free survival 85% and OS 81%) with adjuvant cisplatin 50 mg/m ^2^ intravenously at days 1 and 29 plus volume-directed radiation therapy (45 Gy ± brachytherapy) followed by paclitaxel 175 mg/m ^2^ and carboplatin area under the curve (AUC) 5 every 21 days for four cycles. Given their success and the theoretical benefit of obtaining both local and distant control with combined chemotherapy and radiation, GOG 258 and PORTEC 3 used similar regimens in their experimental arms. Since the last review, the results of GOG 258 have been presented and published in abstract form, and the results of PORTEC 3 have been published ^25, 26^.

GOG 258 compared chemoradiation followed by four cycles of paclitaxel/carboplatin to six cycles of paclitaxel/carboplatin alone ( Table 1) ^25^. The combined arm had fewer vaginal, pelvic, and para-aortic recurrences and more distant recurrences and toxicity; however, there was no improvement in PFS or OS. PORTEC 3 compared chemoradiation plus chemotherapy with whole pelvic radiation alone in patients with high-risk stage I to III endometrial cancer ( Table 1) ^26^. The investigators found that the addition of chemotherapy improved FFS but did not result in a significant improvement in OS. A subgroup analysis of stage III patients showed the greatest FFS advantage (HR 0.66, 95% CI 0.45–0.97) with a non-significant trend toward improved OS (HR 0.71, 95% CI 0.45–1.11). The results of these large phase III studies did not identify a clear best option for adjuvant therapy; however, it may be extrapolated that adjuvant chemotherapy is most important for stage III patients.

In summary, in regard to the latest studies, adjuvant chemotherapy likely has the most benefit in stage III and IV patients. Radiation therapy can be considered for local control. The NCCN recommends systemic therapy with or without vaginal brachytherapy or EBRT with or without vaginal brachytherapy with or without systemic therapy for adjuvant therapy of advanced-stage endometrial cancer ^29^.

### Serous and clear cell endometrial cancers

Uterine serous carcinoma is an aggressive and less common type of endometrial cancer, comprising only 3 to 10% of endometrial cancers but accounting for 39% of endometrial cancer deaths ^30^. Its molecular profile differs from that of type 1 endometrioid histologies; it has a high rate (90%) of TP53 alterations and about a 30% rate of HER2/neu alterations ^31, 32^. High rates of extrauterine spread are common at presentation; 40 to 70% of cases of metastatic disease may be apparent only with comprehensive surgical staging ^33^. The GOG typically requires that tumors classified as serous have more than 50% serous histology if a tumor is of mixed cell type ^34^. However, a study by Boruta *et al*. demonstrated that even serous tumors with a minor component (<10%) had poorer prognosis compared with grade 3 pure endometrioid histology ^35^.

Clear cell endometrial cancers represent about 4% of all uterine tumors and similarly have high rates of occult metastases (40%) and poor survival (40% 5-year survival regardless of stage) ^36^. About 30 to 40% of clear cell carcinomas have TP53 alterations ^32^. They are intermediate in microsatellite instability (MSI) frequency (15%) and phosphatase and tensin homolog (PTEN) alterations (30%) compared with endometrioid (20 to 40% MSI and 10% PTEN alterations) and serous (<5% MSI and 35 to 50% PTEN alterations) carcinomas ^32^. The literature to support definitive recommendations for adjuvant treatment for serous and clear cell uterine cancers is limited because they are relatively rare. Patients with serous and clear cell histology are often included in heterogeneous trials of high-risk uterine cancers. In trials discussed above, GOG 249 included patients with stage I or II serous and clear cell uterine cancer with negative cytology; GOG 258 included stages I or II with positive cytology and stages III or IV; and PORTEC 3 included patients with stages I to III serous or clear cell cancers. It is difficult to draw conclusions regarding treatment since these histologies usually compose less than 20% of the cohort ^6, 24, 26^. Because of their aggressive nature, uterine serous and clear cell carcinomas with any myometrial invasion are often treated with adjuvant chemotherapy. The NCCN guidelines recommend chemotherapy with or without vaginal brachytherapy for IA serous or clear cell endometrial cancers, although they offer observation or EBRT as acceptable alternatives. For IB (or greater) disease, they recommend chemotherapy with or without EBRT with or without vaginal brachytherapy ^29^.

### HER2/neu-positive serous endometrial cancers

Fader *et al*. published their randomized phase II trial of paclitaxel and carboplatin with or without trastuzumab in primary stage III or IV or recurrent HER2/neu-positive uterine serous carcinomas ^37^. They randomly assigned 61 patients and found a median PFS of 12.6 months in the paclitaxel, carboplatin, and trastuzumab arm versus 8.0 months in the paclitaxel and carboplatin alone arm. In the 41 patients with primary advanced-stage disease, the PFS was 17.9 months in the trastuzumab arm versus 9.3 months in the paclitaxel/carboplatin alone arm. In the 17 patients with recurrent disease, PFS was 9.2 months in the trastuzumab arm versus 6 months in the paclitaxel/carboplatin arm. There is a suggestion of an OS advantage in the trastuzumab arm, and the greatest benefit is in the up-front setting, but the data are not yet mature. These preliminary findings are of considerable interest and suggest benefit for up-front HER2/neu tumor profiling to guide adjuvant therapy of this difficult disease.

## Treatment of recurrent endometrial cancers

Systemic recurrence of endometrial cancer is generally considered incurable and is associated with a poor prognosis. A variety of GOG studies have looked at single agents (etoposide, paclitaxel, dactinomycin, liposomal doxorubicin, pyrazoloacridine, topotecan, oxaliplatin, irofulven, flavopiridol, and bevacizumab) and alternating courses of megestrol acetate and tamoxifen in recurrent endometrial cancer, and response rates range from 0 to 31% and 6-month PFS rates from 0 to 43% ^38– 48^. Hormonal therapy is an appealing option in recurrent endometrial cancer given that many endometrial cancers are hormonally driven and the relative lack of toxicity with hormonal therapy. However, response rates and PFS have been disappointing. Given cross-regulation between the estrogen receptor (ER) and PI3K/AKT/mTOR pathway and recent success of combination everolimus and an aromatase inhibitor in aromatase-refractory breast cancer, Slomovitz *et al*. hypothesized that combining everolimus and letrozole would result in improved response rates and PFS ^49, 50^. In their phase II study, they found a clinical benefit rate of 40%, and 23% of patients obtained a complete response ^49^. The authors found that their oral regimen of everolimus 10 mg daily with letrozole 2.5 mg daily was well tolerated, and no patients discontinued the study because of toxicity. Metformin use and CTNNB1 mutations were associated with increased response rates, PFS, and OS, although these findings were not statistically significant. Of note, none of the patients (n = 7) with serous histology had a response. Patients with ER-positive and progesterone receptor (PR)-positive tumors had higher response rates than receptor-negative tumors, although 33% of ER-negative patients and 25% of PR-negative patients had responses. Given these promising results, the investigators evaluated this combined regimen compared with alternating megestrol acetate and tamoxifen in GOG 3007, which was recently published as an abstract ^51^. They reported a 24% response rate in the everolimus/letrozole arm (PFS 6.4 months and OS 20.0 months) and a 22% response rate in the progestin/tamoxifen arm (PFS 3.8 months and OS 16.6 months). Hormonal combined therapies remain appealing options for recurrent endometrial cancer, and biomarkers are needed to better predict which patients will benefit.

## Understanding the molecular biology of endometrial cancer

Advances in our classification of endometrial cancer with molecular profiling may enable us to better stratify risk and recommended therapy. MSI and polymerase-ε (POLE) lead to neoantigens, fragments of proteins expressed by tumor cells that may sensitize the immune system ^52^. The molecular profile of a tumor may better define its prognosis and response to therapy than histology and stage alone. Talhouk *et al*. ^53– 55^, The Cancer Genome Atlas Collaborative (TCGA) ^53– 55^, and the GOG 210 investigators ^53– 55^ have developed different molecular classification schema to better stratify patient risk.

The TCGA project determined four molecularly defined subgroups of endometrial cancer, which yielded excellent prognostic results ^53^. However, the methods required for classification are currently quite expensive and require special handling of the tissue, limiting applicability. Talhouk *et al*. developed a more pragmatic method, the Proactive Molecular Risk Classifier for Endometrial Cancer (ProMisE) classification, to identify molecularly distinct subgroups with a prognostic signature similar to that of the TCGA classification scheme ^54, 56^. The four groups were MMR-deficient (MMR-D), POLE exonuclease domain mutations (POLE EDMs), p53 abnormal, and p53 wild-type. They assessed tumors in a step-wise fashion. The first assessment was based on MMR status, and MMR-D tumors were classified as MMR-D and MMR intact tumors were tested for POLE mutations. POLE mutants were classified as POLE EDM, and POLE wild-type tumors were assessed for p53 status. MMR intact, POLE wild-type tumors were classified as p53 wild-type or p53 abnormal (null/missense mutations). They validated the ProMisE classifier in a large confirmation cohort series and found that POLE EDMs had the best prognosis and p53 mutants the worst. The ProMisE classification system improved outcome prediction compared with European Society for Medical Oncology risk stratification, detecting those at risk for Lynch syndrome and potentially guiding clinical management ^56^.

GOG 210, an NRG Oncology/GOG study of the molecular classification for risk prediction in endometrioid endometrial cancer, was published in *Gynecologic Oncology* in August 2017 ^55^. The investigators sought to develop a classification system for endometrioid endometrial cancers, the most common and potentially challenging endometrial cancer histology to classify. They assessed tumors for mismatch repair (MMR) defects (MSI, MMR IHC, and MLH1 methylation), POLE mutations, and loss of heterozygosity. Using four classifications (MMR deficient, copy number altered (CNA), copy number stable, and POLE mutant), they were able to stratify patients by PFS and OS. Their classification system remained statistically significantly related to clinical outcomes in multivariable analyses. CNA had the worst PFS and cancer-specific survival, whereas the POLE group had the best outcomes. The authors advocate prospective validation of this system and recommend that clinicians consider using it to universally screen for Lynch syndrome and identify additional prognostic information to guide treatment decisions.

Biomarkers and molecular alterations are increasingly being used to guide systemic therapy in the recurrent setting. Table 2 outlines common biomarkers in endometrial cancer, noting differences between type I (grade 1 or 2 endometrioid) and type II (grade 3 endometrioid, serous, clear cell, and carcinosarcoma) endometrial cancers as well as potential targeted therapies ( Table 2).

**Table 2.** Expression of biomarkers in type 1 and type 2 endometrial cancer.

Target	Function	Change	Type 1, percentage	Type 2, percentage	Outcome	Potential targeted therapy
*HER-2/* *neu*	Oncogene	Enhanced expression	Rare	18–80	Poor prognosis, aggressive tumor ^57^	HER2 inhibitors (afatinib ^58^ ^a^, trastuzumab ^37^, and lapatinib ^59^ ^a^)
ER and PR	Transcription factor	Enhanced expression	70–73	19–24	Improved overall survival ^60^	Tamoxifen, megestrol acetate ^38^, medroxyprogesterone acetate ^61^, letrozole ^49, 51^
*p53*	Tumor suppressor	Mutation	5–10	80–90	Poor prognosis ^62^	Anti-VEGF (bevacizumab) ^63– 66^
*PIK3CA*	Oncogene	Mutation	26–90 ^53^	26–36	No association with survival, except exon 9 charge-changing mutations associated with worse survival ^67^	mTOR inhibitor ^68, 69^ (everolimus and temsirolimus ^70^) ± letrozole ^49, 51^
*PTEN*	Tumor suppressor	Mutation, deletion, methylation	35–55	0–11	Poor prognosis ^71– 73^	mTOR inhibitor ^68, 69^ (everolimus ^74^ and temsirolimus ^a^), evaluating combination with olaparib ( ClinicalTrials.gov Identifier: NCT02208375)
*EZH2*	Transcription factor	Enhanced expression	16	36	Poor prognosis, aggressive tumor ^75^	EZH2 inhibitor ^a^ (EPZ-6438, ClinicalTrials.gov Identifier: NCT01897571)
*K-ras*	Oncogene	Mutation	13–26	0–10	Poor prognosis ^76^	MEK inhibitor ^77, 78^ ^a^ (trametinib, cobimetinib, and selumetinib ^b^), GOG-2290 evaluating trametinib ± Akt inhibitor
*MLH1*	DNA repair	Methylation	20–35	0–10	No association with survival ^79^	Checkpoint inhibitor: Pembrolizumab ^52, 80^
PD-L1	Ligand for PD-1, immune checkpoint receptor on tumor-infiltrating lymphocytes	Expression on tumor cells	14–48 ^79, 81^	33 ^82^	Trend toward improved survival ^83^	Checkpoint inhibitor: Pembrolizumab ^84^
MSI	Downstream evidence of deficient DNA mismatch repair system	Polymerase chain reaction amplification of specific microsatellite repeats	33–40 ^79^	2 ^53^	No association with survival ^79^	Checkpoint inhibitor: Pembrolizumab ^52, 80^

First five columns adapted from Engelsen *et al*. ^85^. ^a^Actionability shown in other cancer types ^86^. ^b^Low response rates (6%) shown in *k-ras* unselected population with selumetinib as single agent ^87^. ER, estrogen receptor; EZH2, enhancer of zeste homolog 2; *HER-2*/neu, human epidermal growth factor receptor 2, protoconcogene Neu; *K-*ras, Kirsten RAt sarcoma virus; MLH1, MutL homolog 1; MSI, microsatellite instability; mTOR, mammalian target of rapamycin; PD-1, programmed death 1; PD-L1, programmed death ligand 1; *PIK3CA*, phosphatidylinositol 3-kinase catalytic subunit; PR, progesterone receptor; *PTEN*, phosphatase and tensin homolog; VEGF, vascular endothelial growth factor.

In our practice, we test all primary endometrial cancers with IHC for MMR proteins and, if findings are consistent with Lynch syndrome (absence of staining and no evidence of promoter methylation), proceed to confirmatory testing. In addition, we perform MSI testing if there is a high clinical suspicion for Lynch syndrome even with intact proteins. We perform IHC for HER2/neu status in all serous cancers and confirm overexpression with fluorescence *in situ* hybridization. We have not yet adapted routine molecular classification; however, we optimistically await further results to guide systemic therapy by molecular class. In the recurrent setting, we recommend molecular profiling, testing for immunotherapy biomarkers (programmed death ligand-1 (PD-L1), MSI, and tumor molecular burden), HER2/neu status in serous cancer, and determining ER and PR status.

## Immunotherapy in endometrial cancer

Programmed death 1 (PD-1) is an immune checkpoint receptor expressed on tumor-infiltrating T cells that, when activated by PD-L1, blocks T-cell activation and enables immune evasion ^84, 88– 90^. About one half of endometrial tumors evaluated in KEYNOTE-028, a phase 1b study, were PD-L1–positive ^84^. In this study, investigators evaluated the safety and efficacy of pembrolizumab, an anti–PD-1 monoclonal antibody, in patients with PD-L1–positive tumors. In this heavily pretreated cohort of women, 24 women were enrolled; three patients (13.0%) achieved a partial response and three patients achieved stable disease (median duration of 24.6 weeks). Over half of the patients developed a treatment-related toxicity, of which fatigue, pruritus, pyrexia, and anorexia were the most common. Pembrolizumab was well tolerated overall and resulted in durable response in a subset of patients; however, there remains a need to better identify biomarkers to predict durable response. In 2017, the US Food and Drug Administration (FDA) approved pembrolizumab for all advanced solid tumors that are MSI-high or MMR-D.

Makker *et al*. recently published an interim analysis of their phase 2 study of pembrolizumab plus lenvatinib in biomarker-unselected advanced endometrial cancer ^91^. In the unselected cohort (85% microsatellite stable and 25% PD-L1–positive), 39.6% of patients responded with durable responses (65% had responses greater than 6 months, and median duration of response was not yet achieved) ^91^. This trial led to FDA breakthrough therapy designation for pembrolizumab with lenvatinib in advanced endometrial cancer. The group has started enrollment for a phase 3 randomized clinical trial of pembrolizumab and lenvatinib versus doxorubicin or paclitaxel. Further research is ongoing to identify a combinatorial approach with other immunotherapies, radiation, or systemic treatment in order to augment the effects of immunotherapy in endometrial cancer.

## Future directions and current clinical trials

Although there have been great advances in endometrial cancer in the past few years, there remains much left to do as we continue to refine our understanding and treatment of endometrial cancer. Focus will be on better classifying tumors in order to more appropriately enroll patients in clinical trials specific to that profile. We need to identify better biomarkers to guide personalized therapy as well as to explore combinatorial regimens for targeted therapeutics, hormonal therapy, and immunotherapy. Featured actively recruiting trials include a randomized phase III trial in women with endometrial cancer with high-intermediate risk factors to investigate the role of an integrated clinicopathological and molecular risk profile to guide adjuvant therapy (PORTEC 4-a, ClinicalTrials.gov Identifier: NCT03469674); a phase II trial of paclitaxel, carboplatin, and pembrolizumab in measurable advanced or recurrent endometrial cancer (ClinicalTrials.gov Identifier: NCT02549209); a phase II trial of vaginal cuff brachytherapy followed by adjuvant chemotherapy with carboplatin and dose-dense paclitaxel in patients with high-risk endometrial cancer (ClinicalTrials.gov Identifier: NCT03189446); a phase I study of the Wee I kinase inhibitor AZD1775 in combination with radiotherapy and cisplatin in cervical, upper vaginal, and uterine cancers (ClinicalTrials.gov Identifier: NCT03345784); a randomized phase II study comparing single-agent olaparib, single-agent cediranib, or combination cediranib/olaparib in women with recurrent, persistent, or metastatic endometrial cancer (GY012, ClinicalTrials.gov Identifier: NCT03660826); and multiple trials combining checkpoint inhibitors, immunotherapy, tyrosine kinase inhibitors, poly ADP ribose polymerase (PARP) inhibitors, and/or anti-angiogenic inhibitors (TSR042 Garnet study, UC1805, NRG-GY018, AMANDA study, KEYNOTE-077/ECHO-202, KEYNOTE-775, and ROSCAN).
